# Impact of nutritional status on mortality in geriatric patients with osteoporosis: a retrospective cohort study

**DOI:** 10.1186/s12877-025-06601-5

**Published:** 2025-11-19

**Authors:** Suna Avci, Filiz Demirdag, Zehra Kara, Pelin Degirmenci, Selcan Yenigun, Seyda Bilgin, Hasan Yuksel, Ali Kimiaei, Alper Doventas

**Affiliations:** 1 Department of Geriatric Medicine, Cerrahpasa Faculty of Medicine, Istanbul University-Cerrahpasa, İstanbul Üniversitesi Avcılar Kampüsü, Bağlariçi Cd. No:7, Istanbul, Avcılar 34320 Türkiye; 2 Department of Endocrinology and Metabolic Diseases, Cerrahpasa Faculty of Medicine, Istanbul University- Cerrahpasa, Istanbul, Türkiye; 3 Department of Endocrinology and Metabolic Diseases, Istanbul Basaksehir Cam and Sakura City Hospital, Istanbul, Türkiye; 4 Department of Physical Medicine and Rehabilitation, Bagcilar Training and Research Hospital, Istanbul, Türkiye; 5 Division of Hematology, Department of Internal Medicine, Bahçesehir University Faculty of Medicine, Istanbul, Türkiye

**Keywords:** Osteoporosis, Malnutrition, Fractures, Bone, Mortality, Aged

## Abstract

**Background:**

This study investigated the relationship between mortality and numerous characteristics in geriatric patients with osteoporosis, including nutritional, clinical, anthropometric, and biochemical factors.

**Methods:**

This retrospective cohort comprised geriatric patients diagnosed with osteoporosis between January 2016 and December 2019. Demographics, anthropometrics, comorbidities, laboratory results, bone mineral density (DEXA) analyses, fracture history, and mortality data were obtained. In addition, nutritional status, hand grip strength, and gait speed were measured. The Geriatric Depression Scale (GDS) was applied and nutritional status was assessed using multiple tools: Mini Nutritional Assessment (MNA) Short and Long Forms, Controlling Nutritional Status (CONUT), Geriatric Nutritional Risk Index (GNRI), and Prognostic Nutritional Index (PNI).

**Results:**

Among the 752 patients (mean age 75.3 ± 6.8 years, 69.7% female), the mortality rate was 18.1% during a median follow-up of 55 months. Nutritional assessments significantly predicted both fracture occurrence and mortality. Optimal cut-offs for predicting mortality were: MNA Short Form ≤ 11, MNA Long Form ≤ 24, CONUT > 1.5, GNRI ≤ 105.5, and PNI ≤ 49.9. Independent predictors of mortality included increasing age (*p* < 0.001), male sex (*p* = 0.005), higher body shape index (*p* = 0.006), slower gait speed (*p* = 0.001), MNA Long Form score ≤ 24 (*p* = 0.002), and PNI score ≤ 49.9 (*p* < 0.001).

**Conclusion:**

Malnutrition is strongly associated with higher mortality risk in older osteoporosis patients. Early identification of malnutrition is critical to administering appropriate interventions, and our results show that malnutrition deserves greater attention to understand its broader impacts on outcomes in geriatric patients, particularly those with osteoporosis.

## Introduction

Bone density naturally decreases with age, increasing fracture risks, which can cause considerable morbidity and mortality. Therefore, osteoporosis, which degrades bone density, is a critical disease that can have catastrophic impacts on older individuals [1, 2]. Regardless of fracture development, malnutrition appears to be a factor that contributes to poor clinical outcomes in older osteoporotic patients [3, 4], suggesting an independent relationship with adverse outcomes beyond the effects of fractures and age.

As individuals age, they experience natural declines in appetite and muscle mass [3, 4], limiting the reception of essential nutrients required to maintain tissues and organ systems. Osteoporotic individuals already have a considerable loss in bone mass and mineral density. Therefore, nutritional deficiencies may be associated with a cycle that restricts nutritional intake, increases malnutrition-related risks, and is linked to frailty, morbidity, and mortality [5]. Malnutrition also impairs healing, delays recovery, creates metabolic disorders, and weakens immune response, increasing susceptibility to severe chronic disease states and infections — which are the leading causes of death in this population [4, 6]. Despite these considerable risks, the impact of malnutrition on survival has not been explored in detail among osteoporotic patients. Considering the increase in the prevalence of both conditions in older age and the inter-relationships, our limited knowledge on the topic is a scathing problem that would prevent the development of effective interventions. Addressing malnutrition could improve outcomes in patients with osteoporosis, might prevent osteoporosis development among at-risk individuals, and may break the self-feeding vicious cycle. However, before these hypotheses can be tested, it is critical to procure data concerning the frequency, extent, and impact of malnutrition in vulnerable populations such as geriatric patients with osteoporosis.

We therefore hypothesized that malnutrition could be associated with increased mortality in geriatric patients with osteoporosis, possibly irrespective of fractures and other underlying factors or other outcomes. Considering the advanced age of the population, it was also anticipated that clinical and anthropometric variables, comorbidity burden, and laboratory parameters may also be associated with mortality rates. In this context, our objective was to investigate the relationship between malnutrition and mortality in geriatric patients with osteoporosis, while also attempting to elucidate the impacts of other factors and to identify other clinical, anthropometric, and biochemical factors that had independent associations with mortality risks in this population.

## Materials and methods

### Patients and settings

The study was conducted in the Geriatrics outpatient clinic of Istanbul University-Cerrahpasa, Turkey, between January 2016 and December 2019. All patients diagnosed with osteoporosis who were admitted to the geriatric clinics were examined for inclusion. The International Classification of Disease (ICD) codes were used to filter the diagnoses of patients, and those with any osteoporosis-related definition, including M80.0, M80.1, M80.8, M80.9, M81.0, M81.1, M81.8, and M81.9, were deemed eligible for the investigation. Considering the possible variations in early assessments, we excluded patients with short follow-ups (< 12 months). The follow-up duration was defined as the period between the date the osteoporosis diagnosis was first entered into medical records and the date of data collection or the date of death. Additionally, the absence of laboratory data concerning the variables of interest and incomplete or missing measurements regarding anthropometrics were defined as criteria necessitating exclusion. Patients with conditions that could distort anthropometric parameters, such as decompensated heart failure with unstable hemodynamics or marked lower limb edema, were also excluded, as all participants were ambulatory outpatients.

### Ethics

All data collection processes and patient investigations were designed in accordance with the ethical standards stipulated by Good Clinical Practice guidelines and the Helsinki Declaration and its amendments. Ethics approval was granted by the Clinics Research Ethics Committee of Istanbul University Cerrahpaşa Faculty of Medicine (Decision date: 08.02.2019, decision no: 22517). The requirement for individual informed consent was waived by the Clinics Research Ethics Committee of Istanbul University Cerrahpaşa Faculty of Medicine due to the retrospective nature of the study and the use of anonymized data.

### Data collection and definitions

The patients’ records and radiological images were retrospectively reviewed, and the following data obtained and recorded at the time of diagnosis were collected: age, gender, comorbidity information, anthropometric measurements, hand grip strength and gait speed test results, laboratory findings, bone mineral density (BMD) measured by Dual-Energy X-ray Absorptiometry (DEXA), Geriatric Depression Scale (GDS) scores, nutritional status. Anthropometric and biochemical data were collected at the patient’s first outpatient clinic admission, within one to two weeks of the DEXA assessment. The presence and types of fractures, follow-up durations, and mortality status were obtained from post-diagnosis follow-up records.

#### Anthropometrics

The anthropometric measurements included weight, height, body mass index (BMI), waist circumference, hip circumference, waist-to-hip ratio, upper arm circumference, calf circumference, body shape index, fat mass, and free fat mass. All anthropometric assessments were performed by trained geriatric fellows.

Waist circumference was measured using a tape measure at the midpoint between the lower margin of the last palpable rib and the top of the iliac crest, with the participant standing upright [7]. Hip circumference was measured at the widest part of the hips using a flexible tape measure, while the participant was standing [7]. The waist-to-hip ratio was calculated by dividing the waist circumference by the hip circumference. A waist-to-hip ratio greater than 0.90 for men and greater than 0.85 for women was considered high [7]. Upper arm circumference was measured at the midpoint between the acromion and the olecranon process with the arm relaxed [8]. Calf circumference was measured at the maximum circumference of the calf, while the participant was standing with their weight evenly distributed [8]. Body shape index (A body shape index; ABSI) was calculated using the formula that normalizes waist circumference for height and BMI [9]. Fat mass was assessed via bioelectrical impedance analysis or dual-energy X-ray absorptiometry (DEXA). Free fat mass was calculated by subtracting fat mass from total body weight [9]. Patients with a BMI of 30 or higher were considered obese [10]. Body composition analysis was performed using a TANITA TBF-300 bioelectrical impedance analysis device.

#### Geriatric depression scale

The Geriatric Depression Scale was used to assess depression in older adults. It consists of a series of yes/no questions, with higher scores indicating more severe depressive symptoms [11]. The validity and reliability study of the GDS in Turkish was conducted by Ertan et al. [12]. The GDS was administered by trained geriatric fellows.

#### Sarcopenia diagnosis and related measurements

Hand grip strength was assessed using a Jamar hydraulic hand dynamometer. Patients were seated in a chair with their elbows placed on the table, arms parallel to the floor and flexed at 90°. Each measurement was performed three times, and the highest value was recorded for analysis. It measures muscle strength is an indicator of overall physical health [13]. The gait speed test was performed by instructing participants to walk a distance of 4 m at their usual walking pace. The time taken to complete the distance was recorded using a stopwatch, and gait speed was calculated in meters per second [13].

The diagnosis of sarcopenia was made according to the criteria of the European Working Group on Sarcopenia in Older People 2, which includes low muscle strength (assessed by hand grip strength/Takei© Hand-held Dynamometer, Takei Scientific Instruments Co., Ltd., Japan), low muscle mass (measured via TANITA© TBF 300 bio-impedance analysis, TANITA Corporation, Japan), and/or poor physical performance (evaluated by gait speed) [14]. Sarcopenic obesity was defined as the presence of both sarcopenia and obesity. Obesity was defined according to the World Health Organization criteria, with a BMI ≥ 30 kg/m² considered as the threshold [15].

#### Bone mineral density

Bone mineral density was measured using a Hologic DEXA scanner. The vertebral T-score was obtained by scanning the lumbar spine, while the femoral T-score was measured at the femoral neck. Both scores were used to assess bone mineral density, with results compared to the reference values for a young, healthy adult population.

#### Nutritional assessment tools

The short version of The Mini Nutritional Assessment (MNA) Form score was measured using a questionnaire that included questions regarding dietary habits, recent weight loss, and mobility. It consists of 6 questions, and the total score ranges from 0 to 14, with a score of 12 or above indicating normal nutritional status [16]. The long version of The MNA Form score was measured using a comprehensive questionnaire that evaluates dietary intake, weight history, and physical examination findings. The total score ranges from 0 to 30, with scores below 17 indicating malnutrition [16]. The validity study of the Mini Nutritional Assessment tests in Turkish was conducted by Sarikaya and colleagues [16].

The controlling nutritional status (CONUT) score was derived based on serum albumin levels, total cholesterol, and lymphocyte count. A score of 0 to 1 indicated no malnutrition, while scores of 2 to 4, 5 to 8, and 9 to 12 corresponded to mild, moderate, and severe malnutrition, respectively [1].

The Geriatric Nutritional Risk Index (GNRI) score was calculated using the formula: GNRI = (14.89 x (serum albumin level in g/L) + [41.7 x (actual body weight in kg/ideal body weight in kg). The following formulas, known as Lorenz formulas, were used for calculating ideal weight: height (cm) − 100 - ([height (cm) − 150]/four) for men and height (cm) – 100 - ([height (cm) − 150]/2.5) for women. When the current weight surpassed the ideal body weight, we used the ratio of current weight in kilograms to ideal weight = one. A GNRI score below 82 indicates severe nutritional risk in older individuals [17]. While interpreting GNRI values, potential confounding factors affecting serum albumin levels—such as infections, liver disease, renal dysfunction, and fluid overload/edema—were considered.

The Prognostic Nutritional Index (PNI) score was measured using the formula: PNI = (10 x serum albumin level in g/dL) + (0.005 x total lymphocyte count). Lower PNI scores suggest a poorer nutritional status and a higher risk of complications [2].

All nutritional assessments were administered by trained geriatric fellows.

#### Laboratory measurements

Blood samples were collected after an overnight fast for the analysis of biochemical parameters, including creatinine, calcium, phosphorous, magnesium, albumin, parathyroid hormone (PTH), vitamin D, lymphocyte count, and total cholesterol level. Blood sampling for biochemical analysis was performed at the patient’s first outpatient clinic admission, within one to two weeks of the DEXA assessment. All laboratory measurements were performed in certified laboratories following standardized procedures.

### Statistical analysis

The classical *p* < 0.05 threshold was used to define the significance for all analyses performed on SPSS (v25, IBM). To assess the normality of continuous variables, histograms and Q-Q plots were utilized. Descriptive statistics were presented as mean ± standard deviation for normally distributed continuous variables, median (25th–75th percentiles) for non-normally distributed continuous variables, and frequency (percentage) for categorical variables. The Student’s t-test or the Mann-Whitney U test were employed for the analysis of normally and non-normally distributed continuous variables, respectively. Categorical distributions were subject to testing via chi-square tests or Fisher’s exact test. Receiver operating characteristic (ROC) curve analysis was performed to evaluate the prediction of mortality (thresholds: Youden’s index). Multivariable logistic regression models were created by including significant variables in univariate analysis concerning the examined groups, yielding parameters that were independently involved in mortality.

## Results

A total of 752 patients (524 females; 69.68%), with a mean age of 75.33 ± 6.79 years (min: 65 max:99 years) were included in the study. The mortality rate was 18.09% within the follow-up period (median: 55 months, 50–58). The mortality group was significantly older (*p* < 0.001) and had a greater frequency of males (*p* = 0.026). Notably, survivors had significantly higher weight (*p* < 0.001), BMI (*p* < 0.001), hip circumference (*p* = 0.001), upper arm circumference (*p* < 0.001), calf circumference (*p* < 0.001), fat mass (*p* < 0.001), hand grip strength (*p* < 0.001), and gait speed (*p* < 0.001) compared to the mortality group. In contrast, deceased subjects had significantly higher waist-to-hip ratio (*p* = 0.003), body shape index (*p* < 0.001), obesity percentage (*p* = 0.017), and GDS score (*p* = 0.009) relative to survivors. Furthermore, mortality also appeared to be associated with sarcopenia (*p* = 0.001), atrial fibrillation (*p* = 0.010), heart failure (*p* = 0.002), Parkinson’s disease (*p* = 0.026), dementia (*p* < 0.001), lung disease (*p* = 0.034), and the number of medications used (*p* = 0.014). Laboratory data provided some interesting insights into these relationships, showing lower calcium (*p* = 0.021), albumin (*p* < 0.001), lymphocyte count (*p* = 0.047), and total cholesterol levels (*p* < 0.001) in the mortality group which also had, higher creatinine levels (*p* = 0.001) (Table 1).

**Table 1:** Summary of variables with regard to mortality

	Overall	Mortality		*p*
		No (*n* = 616)	Yes (*n* = 136)	
Age	75.33 ± 6.79	74.21 ± 6.34	80.35 ± 6.50	**< 0.001** ^**†**^
Sex				
Male	228 (30.32%)	176 (28.57%)	52 (38.24%)	**0.026** ^**#**^
Female	524 (69.68%)	440 (71.43%)	84 (61.76%)	
Weight, kg	70.12 ± 13.53	71.18 ± 13.51	65.33 ± 12.60	**< 0.001** ^**†**^
Height, cm	157.41 ± 9.25	157.59 ± 9.07	156.61 ± 10.02	0.264^†^
Body mass index, kg/m^2^	28.35 ± 5.26	28.71 ± 5.24	26.72 ± 5.04	**< 0.001** ^**†**^
Obesity	254 (33.78%)	220 (35.71%)	34 (25.00%)	**0.017** ^**#**^
Waist circumference, cm	99.17 ± 12.29	99.25 ± 12.20	98.80 ± 12.74	0.701^†^
Hip circumference, cm	106.42 ± 10.24	107.01 ± 10.08	103.66 ± 10.58	**0.001** ^**†**^
Waist to hip ratio	0.93 ± 0.09	0.93 ± 0.09	0.95 ± 0.09	**0.003** ^**†**^
High	578 (79.61%)	474 (79.26%)	104 (81.25%)	0.700^#^
Upper arm circumference, cm	28.88 ± 4.03	29.32 ± 3.95	26.87 ± 3.77	**< 0.001** ^**†**^
Calf circumference, cm	36.17 ± 4.48	36.56 ± 4.40	34.37 ± 4.43	**< 0.001** ^**†**^
A body shape index	0.086 ± 0.007	0.085 ± 0.007	0.089 ± 0.007	**< 0.001** ^**†**^
Fat mass, kg	31.19 ± 9.89	32.12 ± 9.33	26.29 ± 11.30	**< 0.001** ^**†**^
Free fat mass, kg	45.60 ± 8.22	45.90 ± 8.21	44.41 ± 8.22	0.096^†^
GDS score	3 (1–7)	3 (1–6)	4 (1–8)	**0.009** ^**‡**^
Hand grip strength, kg	21.45 ± 8.31	21.95 ± 8.34	19.15 ± 7.78	**< 0.001** ^**†**^
Gait speed, m/s	0.97 ± 0.39	1.01 ± 0.37	0.78 ± 0.38	**< 0.001** ^**†**^
Sarcopenia	275 (36.57%)	198 (32.14%)	77 (56.62%)	**< 0.001** ^**#**^
Sarcopenic obese	80 (10.64%)	61 (9.90%)	19 (13.97%)	0.215^#^
Hypertension	546 (72.61%)	447 (72.56%)	99 (72.79%)	0.957^#^
Hyperlipidemia	147 (19.55%)	120 (19.48%)	27 (19.85%)	0.921^#^
Diabetes mellitus	259 (34.44%)	213 (34.58%)	46 (33.82%)	0.867^#^
Atrial fibrillation	29 (3.86%)	18 (2.92%)	11 (8.09%)	**0.010** ^**#**^
Ischemic heart disease	123 (16.36%)	97 (15.75%)	26 (19.12%)	0.336^#^
Heart failure	29 (3.86%)	17 (2.76%)	12 (8.82%)	**0.002** ^**#**^
Hypothyroidism	107 (14.23%)	90 (14.61%)	17 (12.50%)	0.616^#^
Parkinson’s disease	24 (3.19%)	15 (2.44%)	9 (6.62%)	**0.026** ^**§**^
Dementia	35 (4.65%)	17 (2.76%)	18 (13.24%)	**< 0.001** ^**#**^
Depression	165 (21.94%)	141 (22.89%)	24 (17.65%)	0.221^#^
Cerebrovascular disease	28 (3.72%)	22 (3.57%)	6 (4.41%)	0.827^#^
Lung disease	76 (10.11%)	55 (8.93%)	21 (15.44%)	**0.034** ^**#**^
Number of medication use	5 (2–7)	5 (2–7)	5 (3–8)	**0.014** ^**‡**^
Creatinine, mg/dL	0.83 (0.74–1.01)	0.82 (0.73–0.99)	0.90 (0.77–1.14)	**0.001** ^**‡**^
Calcium, mg/dL	9.6 (9.1–10.0)	9.7 (9.2–10)	9.5 (9.0–10.0)	**0.021** ^**‡**^
Phosphor, mg/dL	3.5 (3.0–3.9)	3.5 (3.0–3.9)	3.3 (3.0–3.95)	0.245^‡^
Magnesium, mg/dL	1.90 (1.75–2.04)	1.90 (1.75–2.03)	1.87 (1.75–2.07)	0.985^‡^
Albumin, g/dL	4.3 (4.1–4.5)	4.3 (4.1–4.5)	4.1 (3.8–4.3)	**< 0.001** ^**‡**^
PTH, pg/mL	60.8 (43.3–82.4)	60.7 (44.2–82.4)	61.05 (39.38–82.36)	0.466^‡^
Vitamin D, ng/mL	24.3 (16.2–32.0)	24.2 (16.4–31.9)	24.9 (13.9–35.0)	0.948^‡^
Lymphocyte (x10^3^)	1.98 (1.61–2.46)	2.00 (1.63–2.45)	1.84 (1.50–2.49)	**0.047** ^**‡**^
Total cholesterol, mg/dL	208.26 ± 48.78	212.41 ± 47.91	189.27 ± 48.40	**< 0.001** ^**†**^
DEXA Vertebra t score	−2.32 ± 1.18	−2.41 ± 1.09	−2.13 ± 1.31	0.137^†^
DEXA Femoral t score	−2.42 ± 0.74	−2.38 ± 0.73	−2.51 ± 0.77	0.243^†^
MNA Short form score	12 (11–14)	13 (11–14)	11 (9–13)	**< 0.001** ^**‡**^
MNA Long form score	25 (22–27)	25.5 (22.5–27.25)	23 (17.5–26)	**< 0.001** ^**‡**^
CONUT score	0 (0–1)	0 (0–1)	1 (0–2)	**< 0.001** ^**‡**^
GNRI score	105.73 (101.26–108.71)	105.73 (102.75–108.71)	102.36 (97.00–105.58)	**< 0.001** ^**‡**^
PNI score	53.1 (49.9–55.9)	53.33 (50.7–56.1)	50.05 (46.9–54.5)	**< 0.001** ^**‡**^
Follow-up time, months	55 (50–58)	57 (53–58)	33.5 (19–46)	**< 0.001** ^**‡**^
Fracture history	150 (19.94%)	110 (17.86%)	40 (29.41%)	**0.002** ^**#**^

Descriptive statistics were presented using mean ± standard deviation for normally distributed continuous variables, median (25th percentile − 75th percentile) for non-normally distributed continuous variables, and frequency (percentage) for categorical variables

*CONUT* Controlling nutritional status, *DEXA* Dual x-ray absorptiometry, *GDS* Geriatric depression scale, *GNRI* Geriatric nutritional risk index, *MNA* Mini nutritional assessment, *PNI* Prognostic nutritional index, *PTH* Parathormone

† Student’s t test, ‡ Mann Whitney U test, # Chi-square test, § Fisher’s exact test

**Fig. 1:**
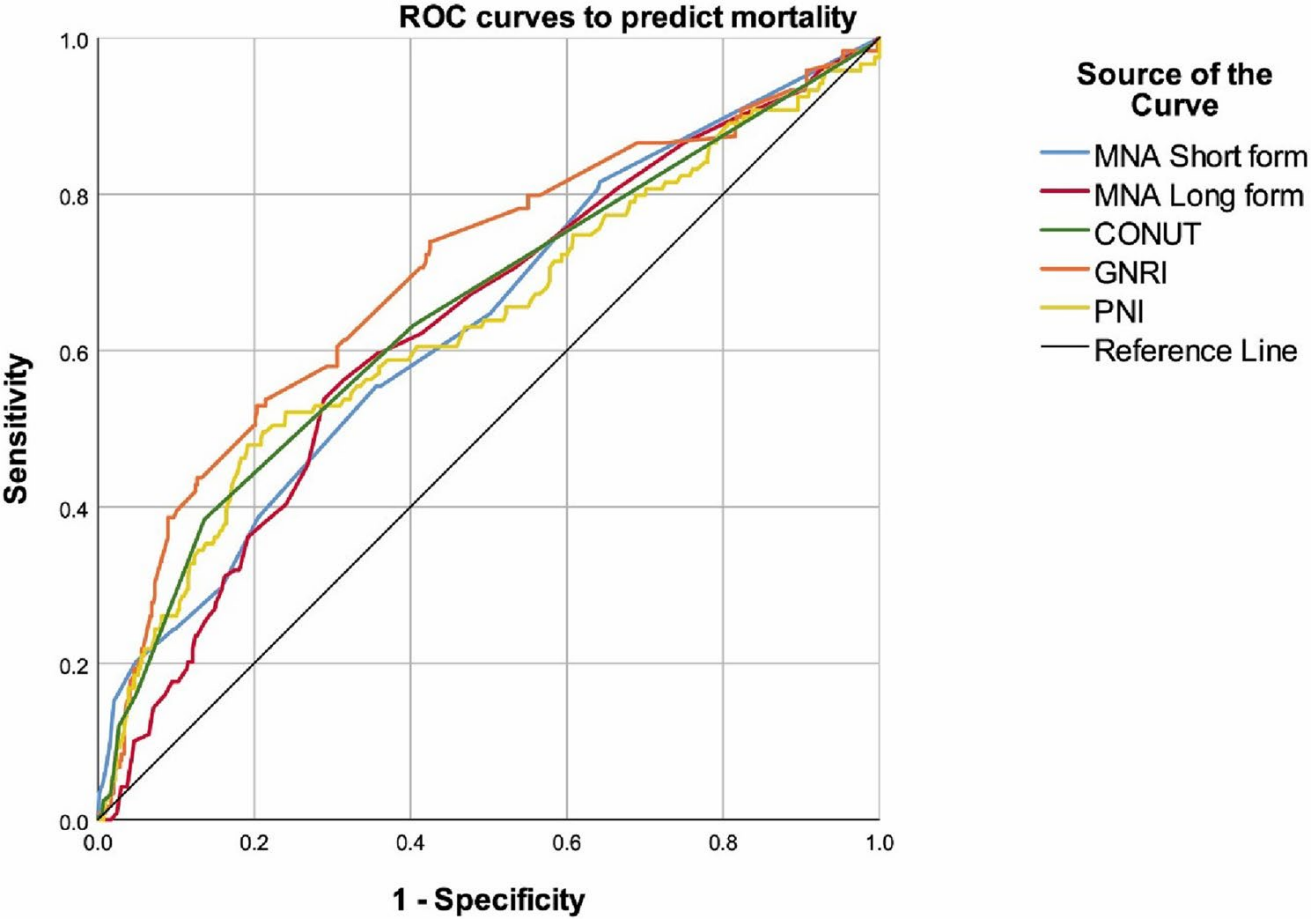
ROC curves of the scores to predict mortality

Nutritional status scales consistently showed worse nutritional status or malnutrition among patients with mortality. The MNA short and long forms (*p* < 0.001 for both), GNRI (*p* < 0.001), and PNI (*p* < 0.001) were significantly lower (worse) in deceased subjects, while CONUT score (*p* < 0.001) was higher (worse) in those with mortality. The frequency of fractures (*p* = 0.002) was also significantly higher in the mortality group (Table 1). Notably, all nutrition assessment tools were successful in predicting mortality and showed largely similar accuracies, albeit with considerable variations in sensitivity and specificity values. CONUT had the highest overall accuracy, closely followed by PNI (Table 2; Fig. 1).

**Table 2:** Performance of scores to predict mortality, ROC curve analysis

	MNA Short form	MNA Long form	CONUT	GNRI	PNI
Cut-off	≤ 11	≤ 24	> 1.5	≤ 105.5	≤ 49.9
Sensitivity	54.20%	59.84%	38.71%	75.00%	50.00%
Specificity	64.72%	64.77%	86.24%	56.99%	80.29%
Accuracy	62.83%	63.90%	77.43%	60.35%	74.64%
PPV	25.18%	26.57%	39.02%	28.57%	36.78%
NPV	86.58%	88.33%	86.08%	90.86%	87.50%
AUC (95% CI)	0.628 (0.574–0.682)	0.633 (0.580–0.687)	0.652 (0.595–0.709)	0.689 (0.636–0.743)	0.643 (0.585–0.700)
p	**< 0.001**	**< 0.001**	**< 0.001**	**< 0.001**	**< 0.001**

*AUC* Area under ROC curve, *CI* Confidence interval, *CONUT* Controlling nutritional status, *GNRI* Geriatric nutritional risk index, *MNA* Mini nutritional assessment, *NPV* Negative predictive value, *PNI* Prognostic nutritional index, *PPV* Positive predictive value, *ROC* Receiver operating characteristic

The analysis of relationships based on the comprehensive dataset yielded several variables that were independently associated with mortality: older age (OR: 1.107, 95% CI: 1.067–1.149, *p* < 0.001), male sex (OR: 2.075, 95% CI: 1.252–3.438, *p* = 0.005), high body shape index (OR: 1.639, 95% CI: 1.149–2.339, *p* = 0.006), and low gait speed (OR: 0.309, 95% CI: 0.156–0.608, *p* = 0.001). Furthermore, among the scales used to assess nutrition, the results showed that a low (≤ 24) MNA long form score (OR: 2.089, 95% CI: 1.296–3.366, *p* = 0.002) and low (≤ 49.9) PNI score (OR: 2.820, 95% CI: 1.731–4.595, *p* < 0.001) were independently associated with mortality. Other variables included in the multivariable model (due to being significant in univariate analysis) were not independently discriminatory for mortality (Table 3).

**Table 3:** Significant factors independently associated with mortality, multivariable logistic regression analysis

	β coefficient	Standard error	*p*	Exp(β)	95% CI for Exp(β)	
Age	0.102	0.019	**< 0.001**	1.107	1.067	1.149
Sex, Male	0.730	0.258	**0.005**	2.075	1.252	3.438
A body shape index (x10^−2^)	0.494	0.181	**0.006**	1.639	1.149	2.339
Gait speed	−1.176	0.346	**0.001**	0.309	0.156	0.608
MNA Long form score, ≤ 24	0.737	0.243	**0.002**	2.089	1.296	3.366
PNI score, ≤ 49.9	1.037	0.249	**< 0.001**	2.820	1.731	4.595
Constant	−13.529	2.008				

Nagelkerke R^2^ = 0.310

*CI* Confidence interval, *MNA* Mini nutritional assessment, *PNI* Prognostic nutritional index

## Discussion

The findings of the present study demonstrated that the MNA Short Form score, MNA Long Form score, CONUT score, GNRI score, and PNI score were significant predictors of mortality in geriatric patients with osteoporosis. Increasing age, male gender, high body shape index, low gait speed, low MNA Long Form score, and low PNI score were independent risk factors for mortality. These findings indicate that identifying and addressing malnutrition could potentially reduce associated mortality rates in this vulnerable patient group, ultimately improving the standard of care and reducing adverse outcomes.

Malnutrition is more prevalent in geriatric individuals and its causes, as well as the outcomes, are multifactorial – and much more severe compared to younger individuals. Malnutrition significantly reduces the regenerative and adaptive capacity in older adults and remains a critical factor influencing successful aging [4]. The reported prevalence of malnutrition varies greatly depending on the definition used. In independently living older adults, malnutrition rates are around 10%, while in hospitalized patients, the prevalence can reach up to 65% [18, 19]. A study on osteoporosis mortality trends in Spain from 1999 to 2015 revealed a significant decline in age-adjusted mortality rates, particularly among women, whose mortality rate decreased faster than men. Notably, women aged 75 and older and men aged 60–64 showed a sustained decline in mortality, with women experiencing the greatest improvements [20]. The improvements may be explained by increased awareness regarding the potential outcomes of malnutrition in the older; however, the discrepancy appears to be limitedly understood. Our study demonstrates that male sex independently increases mortality in this vulnerable population, potentially bridging this gap in knowledge and highlighting the unique needs of males in terms of malnutrition and its treatment.

As mentioned previously, various studies have established the relationship between osteoporosis and malnutrition [4]; however, the extent of the link between malnutrition and mortality risk in older individuals with osteoporosis has remained an unexplored topic. This study, primarily aimed at addressing this knowledge gap, highlights several key factors associated with mortality risk in geriatric patients with osteoporosis. We assessed nutritional status using multiple instruments, including the MNA Short Form, MNA Long Form, CONUT score, GNRI score, and PNI score, all of which were significant predictors of mortality in older patients with osteoporosis. GNRI had the highest AUC and sensitivity, while CONUT had the highest specificity, followed by PNI. The superior predictive performance of GNRI compared to other nutritional tools may be related to its reliance on objective parameters (body weight and serum albumin), its applicability in older populations where body composition and albumin levels are strong survival determinants, and the reduced subjectivity compared to questionnaire-based tools such as MNA. Interestingly, the MNA Long Form scores ≤ 24 and PNI scores ≤ 49.9 were identified as independent risk factors for mortality, despite not having the best predictive capabilities. This is a finding that suggests the need for better tools to detect malnutrition and classify the health risks imposed by different levels of nutritional deficits. However, despite statistical significance, the sensitivity values of these nutritional tools remained relatively low (e.g., CONUT: 38.7%, PNI: 50%), indicating that a considerable proportion of patients at risk of mortality were not identified. This limitation suggests that these tools, while valuable, should ideally be used in combination with clinical variables such as age and comorbidity burden to improve predictive accuracy.

Other independent risk factors for mortality included increasing age, male sex, body shape index, and low gait speed. In a national cohort study from China including 7,700 individuals with osteoporosis, factors such as age, malnutrition in individuals over 65 years old (and specifically in women), diabetes status and nutritional management were found to be associated with all-cause mortality. Individuals with mild malnutrition had a 1.54 times higher risk of mortality, while those with moderate to severe malnutrition had a 2.70 times higher risk compared to individuals with normal nutritional status. Even after adjusting and matching for other factors, malnutrition remained a strong predictor of higher mortality risk [21]. In the this study [21], nutritional status assessment was conducted solely using the Nutritional Risk Index. Bliuc et al. showed that, in older adults with osteoporosis, increased mortality risk following low-trauma fractures (persisting for 5–10 years) was associated with age, quadriceps weakness, subsequent fractures, low BMD, smoking, and reduced physical activity [22]. In a prospective study of 750 women aged 50–94, musculoskeletal deterioration, indicated by low BMD and appendicular lean mass (ALM), was associated with increased all-cause mortality, with mortality risks significantly higher for those with osteopenia and osteoporosis compared to ideal BMD, and for lower ALM categories compared to high ALM, indicating that poor musculoskeletal health independently contributes to mortality risk [23]. In a study involving 15,076 participants from the National Health and Nutrition Examination Survey (NHANES), lower BMD in the femur and lumbar spine was associated with increased all-cause mortality, and osteoporosis was linked to higher mortality risk. Additionally, higher femoral BMD was correlated with decreased cancer and heart disease mortality, showing a sex-specific pattern [24].

Although many previous studies have emphasized that fractures are a significant risk factor for mortality [25, 26], the present study did not find a history of fractures to be an independent risk factor for mortality. Our findings, in line with prior research, indicate that poor nutritional status, as measured by various validated scoring systems (such as GNRI and PNI), serves as a significant predictor of mortality, independent of other risk factors such as age, sex, and physical activity. Additionally, musculoskeletal deterioration —low BMD and decreased ALM— was strongly associated with increased mortality in older patients. The loss of bone mass makes individuals more susceptible to fractures, while reduced muscle mass impairs mobility and balance, further increasing the risk of falls and injury. Although an increased mortality risk is anticipated with the development of fractures and other risk factors that contribute to fracture risks, it is also evident that this relationship does not directly apply to individuals who do not experience fractures. Our study shows that nutritional deficiency, as measured by specifically MNA and PNI, is uniquely associated with mortality in our population of geriatric patients with osteoporosis, rather than fractures. Given these findings, early and targeted interventions that focus on improving nutritional status are critical to improve outcomes in the older people, which adds a novel aspect to the assessment of fracture risks in this population. Adequate protein intake, supplementation of micronutrients such as calcium and vitamin D, and strategies to address malnutrition, may help improve bone and muscle health. Furthermore, exercise programs aimed at enhancing muscle strength, balance, and bone density could significantly reduce fracture risk and improve long-term survival rates in older patients with osteoporosis. Therefore, addressing modifiable risk factors through comprehensive care strategies that revolve around nutrition and physical activity has the potential to mitigate mortality risks observed in this vulnerable population. By intervening early and holistically, healthcare providers can improve both the quality of life and longevity of individuals living with osteoporosis. Nevertheless, our prognostic model did not include functional measures (e.g., activities of daily living, instrumental activities of daily living), social support, or socioeconomic status, all of which are important determinants of survival in the geriatric population. The absence of these variables limits the comprehensiveness of our findings, and future studies should aim to incorporate these parameters to strengthen predictive models.

The present study explores a largely under-researched topic with a comprehensive dataset and includes a large cohort; however, the single-center and retrospective design must still be considered limitations. Although the examined population size and a broad range of variables would allow for generalization to similar populations, the findings may not apply to populations with drastic differences from ours, particularly in terms of eating habits, access to modern medicine, awareness of nutritional requirements, and geographic and lifestyle differences. Also, the exclusion of patients with a follow-up shorter than 1 year may have introduced a selection bias that should be considered when interpreting the results. Additionally, the study focused solely on all-cause mortality to obtain a more holistic view of the impact of malnutrition, which limits the ability to assess cause-specific mortality of osteoporosis. Despite boasting a large dataset, one of the major limitations is the lack of T-score data for each patient. The diagnosis of osteoporosis was based solely on ICD codes, rather than BMD measurements; however, the diagnoses of such conditions are carried out by international guidelines. The absence of BMD data in our dataset drawn from local records does not exclude the possibility that BMD results were obtained in other centers and were used as the basis of the diagnosis. Furthermore, although the total number of medications was available to us, we did not have complete access to medication data, neither for osteoporosis nor comorbid conditions. This prevented the evaluation of the influence of pharmacological treatment on mortality disease progression and mortality. The study lacks data on the lifestyles and physical activity levels of patients, which are important factors in both the progression of osteoporosis and the risk of mortality. Moreover, the follow-up period overlapped with the Covid-19 pandemic, and the associated excess mortality may have affected the generalizability of our findings, particularly with respect to mortality outcomes. Additionally, although we attempted to consider potential confounders such as infections, liver disease, and renal dysfunction when interpreting nutritional indices, residual confounding cannot be excluded, and this remains a limitation of our retrospective design. Lastly, despite statistical significance, the sensitivity values of these nutritional tools remained relatively low (e.g., CONUT: 38.7%, PNI: 50%), indicating that a considerable proportion of patients at risk of mortality were not identified. This limitation suggests that these tools, while valuable, should ideally be used in combination with clinical variables such as age and comorbidity burden to improve predictive accuracy.

## Conclusion

Malnutrition was independently associated with mortality in geriatric patients with osteoporosis. Among the tools evaluated, CONUT demonstrated the highest accuracy, while MNA long form and PNI were also independently predictive despite lower sensitivity and specificity values. Increasing age, male sex, and low physical performance were additional risk factors, whereas fracture history was not independently predictive. These findings underscore the importance of early nutritional and physical health interventions in reducing mortality among older osteoporotic individuals. Future research should focus on integrating nutritional, functional, and social determinants into predictive models to enhance risk stratification in this vulnerable population.

## Data Availability

The datasets used and/or analyzed during the current study are available from the corresponding author on reasonable request.
